# c-Myc Alters Substrate Utilization and O-GlcNAc Protein Posttranslational Modifications without Altering Cardiac Function during Early Aortic Constriction

**DOI:** 10.1371/journal.pone.0135262

**Published:** 2015-08-12

**Authors:** Dolena Ledee, Lincoln Smith, Margaret Bruce, Masaki Kajimoto, Nancy Isern, Michael A. Portman, Aaron K. Olson

**Affiliations:** 1 Seattle Children’s Research Institute, Seattle, WA, United States of America; 2 Department of Pediatrics, Division of Critical Care Medicine, University of Washington, Seattle, Washington, United States of America; 3 Environmental Molecular Sciences Laboratory (EMSL), Pacific Northwest National Laboratory, Richland, WA, United States of America; 4 Department of Pediatrics, Division of Cardiology, University of Washington, Seattle, Washington, United States of America; Université catholique de Louvain, BELGIUM

## Abstract

Hypertrophic stimuli cause transcription of the proto-oncogene c-Myc (Myc). Prior work showed that myocardial knockout of c-Myc (Myc) attenuated hypertrophy and decreased expression of metabolic genes after aortic constriction. Accordingly, we assessed the interplay between Myc, substrate oxidation and cardiac function during early pressure overload hypertrophy. Mice with cardiac specific, inducible Myc knockout (MycKO-TAC) and non-transgenic littermates (Cont-TAC) were subjected to transverse aortic constriction (TAC; n = 7/group). Additional groups underwent sham surgery (Cont-Sham and MycKO-Sham, n = 5 per group). After two weeks, function was measured in isolated working hearts along with substrate fractional contributions to the citric acid cycle by using perfusate with ^13^C labeled mixed fatty acids, lactate, ketone bodies and unlabeled glucose and insulin. Cardiac function was similar between groups after TAC although +dP/dT and -dP/dT trended towards improvement in MycKO-TAC versus Cont-TAC. In sham hearts, Myc knockout did not affect cardiac function or substrate preferences for the citric acid cycle. However, Myc knockout altered fractional contributions during TAC. The unlabeled fractional contribution increased in MycKO-TAC versus Cont-TAC, whereas ketone and free fatty acid fractional contributions decreased. Additionally, protein posttranslational modifications by O-GlcNAc were significantly greater in Cont-TAC versus both Cont-Sham and MycKO-TAC. In conclusion, Myc alters substrate preferences for the citric acid cycle during early pressure overload hypertrophy without negatively affecting cardiac function. Myc also affects protein posttranslational modifications by O-GlcNAc during hypertrophy, which may regulate Myc-induced metabolic changes.

## Introduction

The proto-oncogene c-Myc (Myc) is an important regulator of cardiac hypertrophy [1–9]. Although the mature heart does not express Myc during normal physiological conditions, hypertrophic or stressful stimuli cause its myocardial transcription [9]. In a transgenic mouse model, cardiac specific Myc induction caused hypertrophy with maintained function [8]. In contrast, inducible knockout of myocardial Myc reduced hypertrophy caused by transverse aortic constriction (TAC) in mice [9,10]. The mechanisms by which Myc modifies the hypertrophic response still requires clarification.

In prior experiments, we demonstrated that myocardial Myc induction in mice promoted a shift in oxidative substrate preferences from carbohydrates to fatty acids [11]. This finding contrasts with other models of hypertrophy, which paradoxically promote glucose utilization relative to fatty acids [12]. Some investigators have suggested that these shifts in myocardial metabolism affect cardiac function [12–14]. Thus, metabolic regulation may serve as a mechanism for Myc promotion of functional compensation during hypertrophy. Further, we found that the shift towards fatty acid oxidation during Myc induction was accompanied by increased protein posttranslational modifications by beta-O-linked *N*-acetylglucosamine (O-GlcNAc) [11]. This modification occurs as a result of glucose shunting into the hexoseamine biosynthetic pathway. Myc induction did not alter expression or phosphorylation state for typical controllers of substrate oxidation, such as PPARα and its downstream targets. As prior evidence shows that acute O-GlcNAc augmentation promotes myocardial fatty acid oxidation [15], these modifications may serve as the metabolic regulators during Myc induction.

However, it is unknown whether Myc similarly regulates these metabolic pathways during stress-induced hypertrophy. Accordingly, we assessed the interplay between Myc and these metabolic processes during early pressure overload hypertrophy induced by TAC. We used a previously described mouse model harboring a cardiac selective inducible Myc knockout [9,10] to determine the relationships between cardiac hypertrophy, substrate metabolism, and O-GlcNAcylation in this model. Our metabolic evaluations using ^13^Carbon (^13^C) substrate labeling and isotopomer analysis determined the fractional contribution of acetyl-CoA to the citric acid cycle from multiple substrates supplied to the heart simultaneously in physiologic concentrations.

## Materials and Methods

### Ethics statement

This investigation conforms to the Guide for the Care and Use of Laboratory Animals published by the National Institute of Health (NIH Pub. No. 85–23, revised 1996) and were reviewed and approved by the Office of Animal Care at Seattle Children’s Research Institute.

### Animals

Cardiac specific, inducible Myc knockout mice on a FVB background were kindly provided by Dr. Robb MacLellan and have been previously described [9,10]. All experiments utilized male mice between 4–6 months of age. To induce excisional recombination of Myc (in MerCreMer(MCM); Myc^fl/fl^), mice were treated with 1 mg 4-hydroxytamoxifen (4-OHT) in peanut oil for twelve days (hereafter referred to MycKO). Non-MCM; Myc^fl/fl^ littermates also received 4-OHT prior to surgery and served as controls (Cont). Although higher dosing of 4-OHT was noted to cause a transient cardiomyopathy in MCM mice [16], our 4-OHT dosing did not cause any adverse effects on echocardiographically determined cardiac function (data not shown). Nevertheless, we waited twelve days or more after completing 4-OHT injections before surgery.

### Transverse aortic constriction surgery (TAC)

TAC was used to create pressure overload cardiac hypertrophy. The surgery was performed as described by deAlmeida et al [17]. Briefly, the mice were initially anesthetized with 3% isoflurane in 100% O_2_ at a flow of 1 LPM and then maintained with 1.5% isoflurane for the duration of the surgery. A midline sternotomy was performed to expose the aorta which was subsequently constricted with a 7–0 silk suture tied against a 25 gauge blunt needle. Sham mice (Sham) had their aorta mobilized, but no suture tightened. For pain control, the mice received intraperitoneal buprenorphine (0.05–0.1 mg/kg IP) every 8–12 hours for two days. To correlate with the prior work in this transgenic mouse [9,10], we performed our experiments after two weeks of TAC. The hearts were utilized for either metabolic (working heart perfusions) or molecular studies. The experimental groups for this study are MycKO-TAC, Cont-TAC, MycKO-Sham and Cont-Sham. Our previous study showed that 4-OHT does not affect substrate preferences or function in unstressed, non-hypertrophic hearts [11], therefore Cont-Sham mice did not receive 4OH-TAM. Similar to previous studies [9,10], Myc knockout did not affect survival after TAC.

### Echocardiograms

Serial echocardiograms were performed prior to and after two weeks of TAC. Mice were initially sedated with 3% isoflurane in O_2_ at a flow of 1 LPM and placed in a supine position at which time the isoflurane is reduced to 1.5% administered via a small nose cone. ECG leads were placed for simultaneous ECG monitoring during image acquisition. Echocardiographic images were performed with a Vevo 2100 machine using a MS250 or MS550 transducers (VisualSonics, Inc, Toronto, Canada). M-Mode measurements at the midpapillary level of the left ventricle (LV) were performed at end-diastole (LVEDD) and end-systole (LVESD) to determine LV function via the fractional shortening [(LVEDD-LVESD)/LVEDD * 100] in a parasternal short axis mode for at least three heart beats. The pulsed Doppler velocity was measured across the aortic constriction site. This velocity serves as a non-invasive estimate of the peak instantaneous pressure gradient across the constriction site and is calculated by the modified Bernoulli equation (Δ pressure gradient = 4*velocity^2^). Clinically, the calculated mean pressure gradient is often a better estimate of the pressure gradient measured invasively from cardiac catheterization. The mean pressure gradient was estimated by dividing the modified Bernoulli equation in half.

### Isolated working heart preparation

Experiments were performed as previously described with changes noted below [11,18]. The primary difference is that the sham and TAC mice (Cont-TAC and MycKO-TAC) were perfused at different afterloads. *In vivo* afterload increases with aortic constriction. To simulate *in vivo* conditions, the MycKO-TAC and Cont-TAC hearts were perfused at a higher afterload than the Sham hearts. We felt that this strategy would more accurately reflect *in vivo* substrate utilization patterns than perfusing sham and TAC hearts at the same afterload. Additionally, studies in Langendorff (retrograde aortic) mouse heart perfusions have shown that different perfusion pressures are needed to achieve similar coronary flows between aortic constricted and sham hearts [19,20]. Because of the different loading conditions, we did not directly compare function between Sham and TAC hearts. Briefly, mice were heparinized (5000 U/kg ip) and anesthetized with a mixture of ketamine (90 mg/kg) and xylazine (10 mg/kg). After adequate sedation, the aorta was rapidly cannulated, heart excised and placed on the perfusion system. The hearts were initially perfused in a Langendorff manner (70 mmHg perfusion pressure) with physiological salt solution (PSS), pH 7.4, containing (in mmol/l) 118.0 NaCl, 25.0 NaHCO_3_, 4.7 KCl, 1.23 MgSO_4_, 1.2 NaH_2_PO_4_, 5.5 D-glucose, and 1.2 CaCl_2_. During retrograde perfusion, the left atrium was cannulated and connected to the preload reservoir. Spontaneously beating hearts were then switched to the antegrade work-performing mode with perfusion into the left atrium of semi-recirculating PSS containing the following ^13^C-labeled substrates in addition to unlabeled glucose (5.5 mmol/l): 1,3-[^13^C]acetoacetic acid (referred to as ketone bodies, 0.17 mmol/l), L-lactic-3-[^13^C]acid (Lactate, 1.2 mmol/l), and U-[^13^C]-long-chain mixed free fatty acids (free fatty acids or FFA, 0.35 mmol/l) bound to 0.75% (wt/vol) delipidated bovine serum albumin reconstituted with deionized water. Insulin (50 microunits/ml) was included in the perfusion solutions. To establish the origin of the unlabeled fraction in the aortic constricted mice, 2 hearts each from Cont-TAC and MycKO-TAC were perfused with unlabeled lactate (1.2 mmol/l), 1-[^13^C]glucose (5.5 mmol/l), 1,3-[^13^C]ACAC (0.17 mmol/l), U-[^13^C]free fatty acids (0.35 mmol/l). The hearts subjected to TAC (Cont-TAC, MycKO-TAC) were initially stabilized at 50 mmHg afterload and 15 mmHg preload for 10 minutes before acutely increasing afterload to 80 mmHg. Sham mice were perfused with standard loading conditions of our laboratory consisting of a 12 mmHg preload and a 50 mmHg afterload. An SPR-PV-Catheter (SPR-869 or -839 Millar Pressure-Volume Systems, Millar Instruments, Inc, Houston, TX) was inserted into the left ventricle through the apex for continuous measurement of left ventricular pressure (LVP). Only aortic outflow that bypasses the coronary arteries is recirculated. Left atrial inflow was measured with a flow probe (T403; Transonic Systems, Ithaca, NY) and aortic (not including coronary flow) flow was measured via 30 second timed collections. Coronary flow was calculated as the difference between left atrial inflow and aortic flow although this measurement is affected by perfusate leak from the left atrium. Every 10 minutes, left atrial influent and coronary effluent was collected for determination of PO_2_, PCO_2_, and pH with an ABL800 blood gas analyzer (Radiometer, Copenhagen, Denmark). The coronary effluent is collected as it drips from the heart. Although we attempt to limit effluent exposure time to the atmosphere, some atmospheric gas exchange likely occurs which would affect the oxygen tension levels and calculations based upon this measurement (for example MVO_2_, and calculated estimated flux). Continuously recorded parameters are left ventricular (LV) pressure (mmHg), HR (beats/min), and rate of LV contraction and relaxation (±dP/dt, mmHg/s). Myocardial oxygen consumption (MVO_2_) was calculated as MVO_2_ = CF x [(Pa_O2_—Pv_O2_) x (*c*/760)] x heart weight, where CF is coronary flow (ml·min^−1^g wet weight^−1^), (Pa_O2_—Pv_O2_) is the difference in the partial pressure of oxygen (P_O2_, mmHg) between perfusate and coronary effluent, and *c* is the Bunsen solubility coefficient of O2 in perfusate at 37°C (22.7 μl O_2_·atm^-1^·ml^-1^). Cardiac work was calculated as the cardiac output times the developed pressure. Cardiac efficiency was defined as cardiac work/MVO_2_.

### ^13^C Magnetic Resonance Spectroscopy (MRS) and isotopomer analyses to determine substrate utilization for the citric acid cycle

Myocardial tissue was extracted as previously described [11,18]. Substrate metabolism was determined by using ^13^C-labeled substrates in combination with NMR spectroscopy. Glutamate isotopomer analyses provide fractional contribution of acetyl-CoA to the citric acid cycle from up to three differentially labeled substrates as well as the unlabeled components.

Lyophilized heart extracts were dissolved in 99.8% D2O for decoupled ^13^C NMR spectral acquisition. NMR free-induction decays (FIDs) were acquired on a Varian Direct Drive (VNMRS) 600 MHz spectrometer (Varian Inc., Palo Alto, CA) equipped with a Dell Precision 390 Linux workstation running VNMRJ 2.2C. The spectrometer system was outfitted with a Varian triple resonance salt-tolerant cold probe with a cold carbon preamplifier. A Varian standard one dimensional carbon direct observe sequence with proton decoupling was used to collect data on each sample. Final spectra were accumulations of 4800 individual FIDs. Each FID was induced using a nonselective, 45-degree excitation pulse (7.05 us @ 58 dB), with an acquisition time of 1.3 seconds, a recycle delay of 3 seconds, and a spectral width of 224.1 ppm.

FIDs were baseline corrected, zero-filled, and Fourier transformed. Labeled carbon resonances of glutamate were integrated with the Lorentzian peak fitting subroutine in the acquisition program (NUTS, Acorn NMR, Livermore, CA). The individual integral values were used as starting parameters for the citric acid cycle analysis fitting algorithm tcaCALC, kindly provided by Drs. Malloy and Jeffrey [21]. This algorithm provided the fractional contributions for each substrate in the acetyl-CoA pool entering citric acid cycle. The estimated total flux for the citric acid cycle and oxidative flux for individual substrates were calculated from MVo_2_ and the stoichiometric relationships between oxygen consumption and citrate formation from the various substrates as previously described [21,22].

### Immunoblotting

Western blots were performed as previously described on freshly isolated hearts (i.e. separate from the perfused hearts) [11,18]. Briefly, fifty micrograms of total protein extract from mouse heart tissue was separated electrophoretically and transferred to PVDF membrane. The membranes were probed with the following antibodies: pyruvate kinase M2 isoform (PKM2, #4053), lactate dehydrogenase A (LDHA, #2012), acetyl-CoA carboxylase (ACC, #3662), phospho-acetyl-CoA carboxylase, (p-ACC, Ser 79, #3661), and pyruvate dehydrogenase (PDH, #2784) were purchased from Cell Signaling Technology, Inc. (Danvers, MA); hexokinase II (HXKII, sc-6521), glutamine-fructose-6-phosphate transaminase 2 (GFAT2, sc-134710), O-GlcNAcase (OGA, sc-135093) and peroxisome proliferator-activated receptor gamma coactivator 1 alpha (PGC-1α, sc-13067) were purchased from Santa Cruz Biotechnology, Inc. (Santa Cruz, CA); O-GlcNAc transferase (OGT, O6264) from Sigma-Aldrich Corp. (St. Louis, MO); phospho- pyruvate dehydrogenase (p-PDH, Ser293, #ABS204) was purchased from EMD Millipore Corporation (Billerica, MA);malonyl CoA-carboxylase (MCD, 15265-1-AP) was purchased from Proteintech (Chicago, IL); glucose transporter 4 (Glut4, GTX88031) was purchased from GeneTex (Irvine, CA). Muscle carnitine palmitoyltransferase 1 (M-CPT1) and pyruvate dehydrogenase kinase (PDK)2 and PDK4 were obtained as a personal gift from Gebre Woldegiorgis (Oregon Health Sciences University, Beaverton, OR) and Robert Harris (Indiana University School of Medicine, Indianapolis, IN). Total protein O-GlcNAc levels were determined using an antibody (RL-2) from Abcam (Cambridge, MA) on freshly isolated protein. Western blots were visualized with enhanced chemiluminescence upon exposure to Kodak BioMax Light ML-1 film. Membranes were stripped by washing for 30 min with 100 mM dithiothreitol, 2% (wt/vol) SDS, 62.5 mM Tris·HCl, pH 6.7, at 70°C, followed by three 10-min washes with TBS for additional antibody analysis. Immunoblots of proteins without a phosphorylated form were normalized to total protein staining in the molecular weight region around the band of interest by Thermo Scientific Pierce Reversible Protein Stain Kit for PVDF Membranes (Thermo Scientific, Rockford, IL), which are shown. Phosphorylated proteins were normalized to their appropriate total protein.

### Statistical analysis

Reported values are means ± standard error (SE) in figures and text. Data were analyzed with two-way unpaired t-test or a single factor ANOVA (multiple comparisons). If significance was identified between the groups by ANOVA (P-value < 0.05); then a two-way unpaired t-test was performed. Criterion for significance was p < 0.05 for all comparisons.

## Results

### Morphometric and echocardiographic data

Prior echocardiogram studies demonstrated that Myc knockout did not affect *in vivo* systolic function measured during TAC [9]. We similarly performed echocardiographic assessment to corroborate this functional result, as well as, to measure the degree of aortic constriction in our experimental groups (Table 1). Myc knockout did not affect left ventricular size or systolic function prior to surgery (Cont baseline and MycKO baseline). Of note, baseline studies represent normal physiologic condition. TAC resulted in comparable aortic constriction velocities between the groups with estimated mean pressure gradients of 44±1 mmHg in Cont-TAC and 43±2 in MycKO-TAC. There were no *in vivo* systolic functional differences between MycKO-TAC and Cont-TAC hearts after TAC. Compared to baseline (pre-TAC), ejection and shortening fractions were unchanged by TAC in both groups. Left ventricular end diastolic diameter decreased in both groups after TAC whereas end systolic diameter declined in Cont-TAC only. The diameter changes are likely due to the development of hypertrophy.

**Table 1:** Echocardiographic analysis of the left ventricle and transverse aorta at baseline and after two weeks of transverse aortic constriction (TAC).

	Cont Baseline (n = 8)	Cont-TAC (n = 8)	MycKO Baseline (n = 8)	MycKO-TAC (n = 8)
**HR (BPM)**	512±24	540±12	507±19	541±12
**TAC velocity (m/sec)**	N/A	4.8±0.2	N/A	4.6±0.1
**EDD, mm**	4.1±0.1	3.9±0.1*	4.2±0.1	3.9±0.1*
**ESD, mm**	2.9±0.1	2.6±0.1*	2.9±0.1	2.7±0.1
**FS, %**	29±2	32±2	31±1	30±1
**ESD, mm**	55±3	61±3	59±2	58±2

HR, heart rate; BPM, beats per minute; EDD, left ventricular end diastolic diameter; ESD, left ventricular end systolic diameter; FS, left ventricular per cent fractional shortening; EF, left ventricular per cent ejection fraction; N/A, not applicable. Values are means±SEM.

* = p<0.05 versus baseline in the same group.

In prior studies, Myc knockout during aortic constriction attenuated the ratio of heart weight to tibial length, reduced cardiomyocyte size, and decreased ANP levels [9,10]. To confirm that Myc reduces hypertrophy in our experiments, we measured the heart weight to tibial length ratio. Heart weight to tibial length was reduced in MycKO-TAC versus Cont-TAC (p<0.05, Table 2,). Given that the estimated mean pressure gradients across the aortic constrictions were similar between the TAC groups, the differences in heart mass were not from different loading conditions. Cont-Sham hearts were significantly smaller (p<0.05) than either TAC group. Liver and lung wet weight (normalized to tibial length) were similar among all groups. MycKO-Sham hearts were not measured because Myc inhibition does not affect cardiomyocyte size or cardiac mass during normal physiologic conditions [9,10].

**Table 2:** Morphometric measurements of heart weight (heart), liver weight (liver), and lung weight (lung) normalized to tibial length (TL).

	Cont-Sham (n = 5)	Cont-TAC (n = 8)	MycKO-TAC (n = 8)
**Heart/TL (mg/mm)**	6.3±0.2	8.3±0.5*	7.2±0.2* ^,^ ^#^
**Liver/TL (mg/mm)**	83±5	78±4	76±2
**Lung/TL (mg/mm)**	8.6±0.1	8.5±0.4	8.3±0.6

All weights are on wet organs. Values are means±SEM.

* = p<0.05 in the indicated group versus Sham;

^#^ = p<0.05 MycKO-TAC versus Cont-TAC.

### Cardiac function during the working heart experiments

*Ex vivo* functional assessments were made during the working heart perfusions. All reported values are after 20 minutes of stable left atrial infusion.

To simulate *in vivo* conditions, Cont-TAC and MycKO-TAC hearts were perfused at high afterload (80 mmHg) whereas Cont-Sham and MycKO-Sham hearts were subjected to standard afterload (50 mmHg). The different afterloads affect functional parameters; therefore we did not compare sham heart function to TAC. *Ex vivo* cardiac function was similar between Cont-Sham and Cont-MycKO (Table 3). In the aortic constricted hearts, there was a trend towards superior +dP/dT_max_ (p = 0.08) and-dP/dT_min_ (p = 0.05) in MycKO-TAC hearts compared to Cont-TAC although other functional parameters were similar (Table 4).

**Table 3:** Functional measurement during isolated working heart perfusions with ^13^C-labeled substrates at standard afterload (50 mmHg) in Cont-Sham and MycKO-Sham mice.

	Cont-Sham (n = 5)	MycKO-Sham (n = 5)
**Heart rate (BPM)**	396±17	410±8
**+dP/dT**_**max**_ **(mmHg/sec)**	4728±284	4466±197
**-dP/dT**_**min**_ **(mmHg/sec)**	-4085±277	-4030±242
Power ((ml/min)*mmHg)	1278±130	1423±167
**Aortic flow (ml/min)**	11.4±0.7	13.0±1.4
**Coronary flow (ml/min)**	4.2±0.3	4.1±0.3
**MVO**_**2**_ **(μmol/g wet weight/min)**	8.0±0.4	7.1±0.5

MVO_2_, myocardial oxygen consumption. Values are means±SEM.

**Table 4:** Functional measurement during isolated working heart perfusions with ^13^C-labeled substrates at high afterload (80 mmHg) in mice subjected to transverse aortic constriction (TAC).

	Cont-TAC (n = 7)	MycKO-TAC (n = 7)
**Body Weight (g)**	30.6±0.9	30.8±1.1
**Heart rate (BPM)**	411±14	396±9
**+dP/dT**_**max**_ **(mmHg/sec)**	5327±415	6158±202 (p = 0.08)
**-dP/dT**_**min**_ **(mmHg/sec)**	-4016±261	-4681±205 (p = 0.05)
Power ((ml/min)*mmHg)	827±99	864±51
**Aortic flow (ml/min)**	5.4±0.6	5.4±0.5
**Coronary flow (ml/min)**	3.9±0.3	3.3±0.2
**MVO**_**2**_ **(μmol/g wet weight/min)**	5.8±0.5	5.2±0.5
Efficiency (ml*mmHg/μmol O_**2**_ **/g)**	144±12	172±14

MVO_2_, myocardial oxygen consumption. Cardiac efficiency is defined as cardiac power/oxygen consumption. Values are means±SEM.

### Fractional contribution of acetyl-CoA to the citric acid cycle

**Fig 1:**
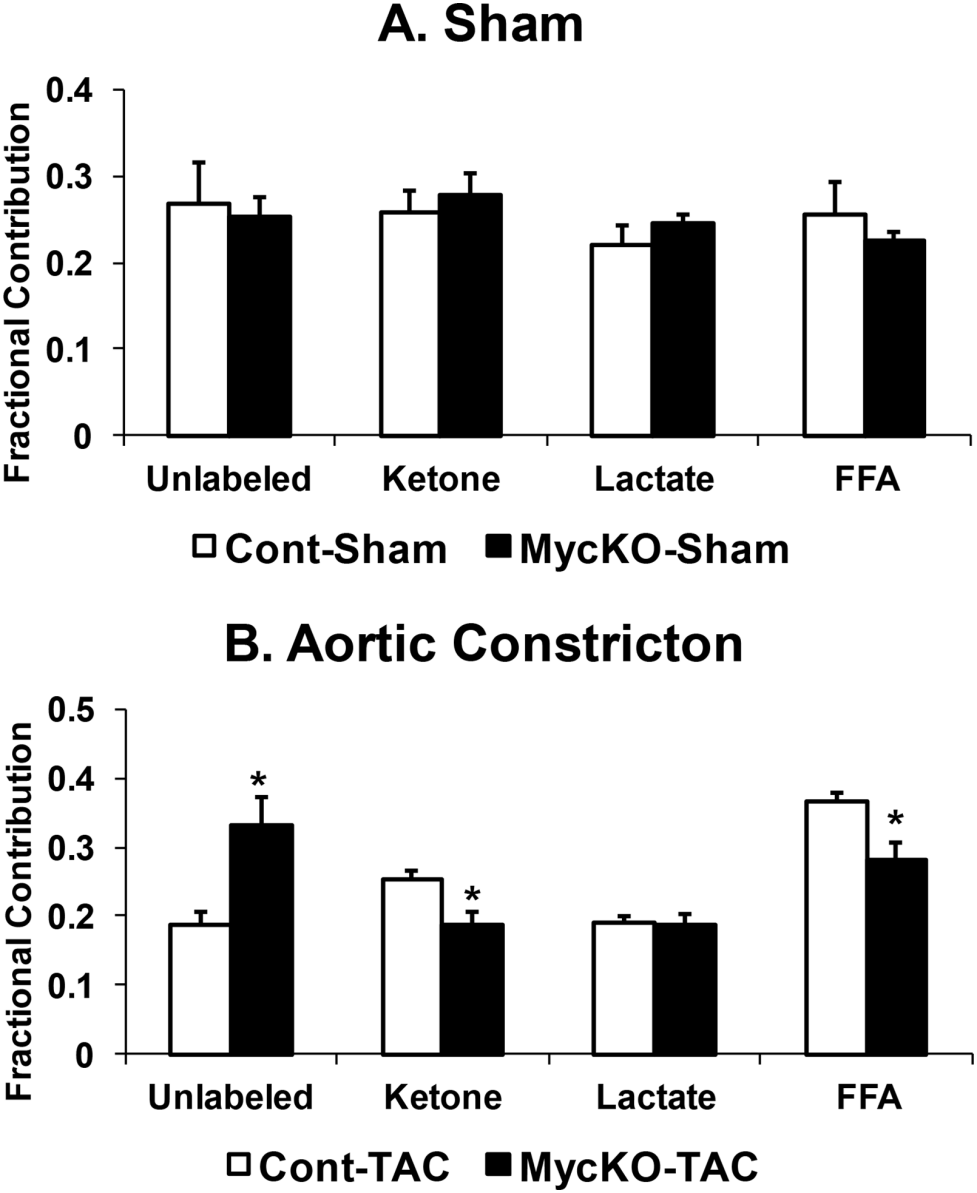
Fractional contribution to the citric acid cycle for unlabeled substrates (unlabeled), acetoacetate as ketone bodies (ketone), lactate, and free fatty acids (FFA). A. Sham mice fractional contributions. B. Fractional contributions in aortic constricted mice. Values are means±SEM. n = 5 Cont-Sham, n = 5 MycKO-Sham, n = 7 Cont-TAC, n = 7 MycKO-TAC. * = p<0.05 between indicated groups.

Next, we determined the fractional contribution of acetyl-CoA to the citric acid cycle for each studied substrate. In sham hearts, fractional contributions were unaffected by Myc knockout (Cont-Sham versus MycKO-Sham, Fig 1A). However, Myc knockout during aortic constriction altered fractional contributions (p<0.05, Fig 1B, S1 Table). Unlabeled fractional contribution increased in MycKO-TAC versus Cont-TAC, whereas ketone and free fatty acid fractional contributions decreased. The unlabeled fraction is composed of exogenous glucose, endogenous glycogen, and endogenous triglycerides. Perfusions with 1-[^13^C] glucose (described in Materials and Methods) gave an average calculated glucose fractional contribution of 0.135±0.005 in Cont-TAC and 0.22±0.02 in MycKO-TAC, which indicates that the majority of the unlabeled fraction was from exogenous glucose in the TAC groups.

**Fig 2:**
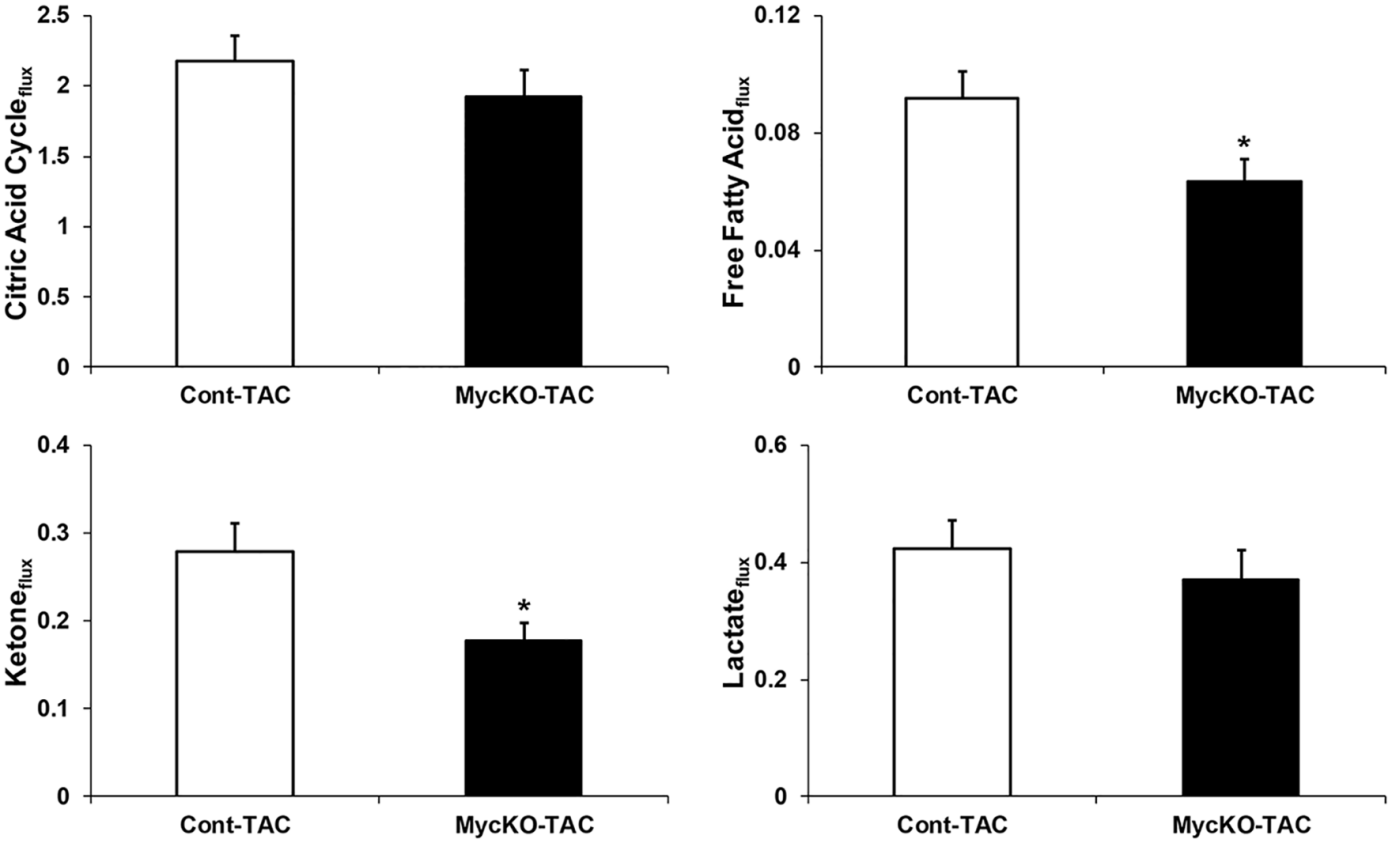
Calculated estimations of flux rates for total citric acid cycle and individual substrates. Units are μmol/g/min. Acetoacetate was used for ketones. Values are means±SEM. n = 6 Cont-TAC, n = 7 MycKO-TAC. * = p<0.05 between groups.

The fractional contributions and oxygen consumption data can be used for calculations estimating citric acid cycle and substrate fluxes [21,22]. These results are shown in Fig 2 and S1 Table. The overall estimated citric acid cycle flux was similar between Cont-TAC and MycKO-TAC The calculated fluxes for both free fatty acids and ketones decreased in MycKO-TAC versus Cont-TAC (p<0.05). Cont-Sham and MycKO-Sham estimated fluxes were similar (data not shown).

### Protein expression of metabolic enzymes

**Fig 3:**
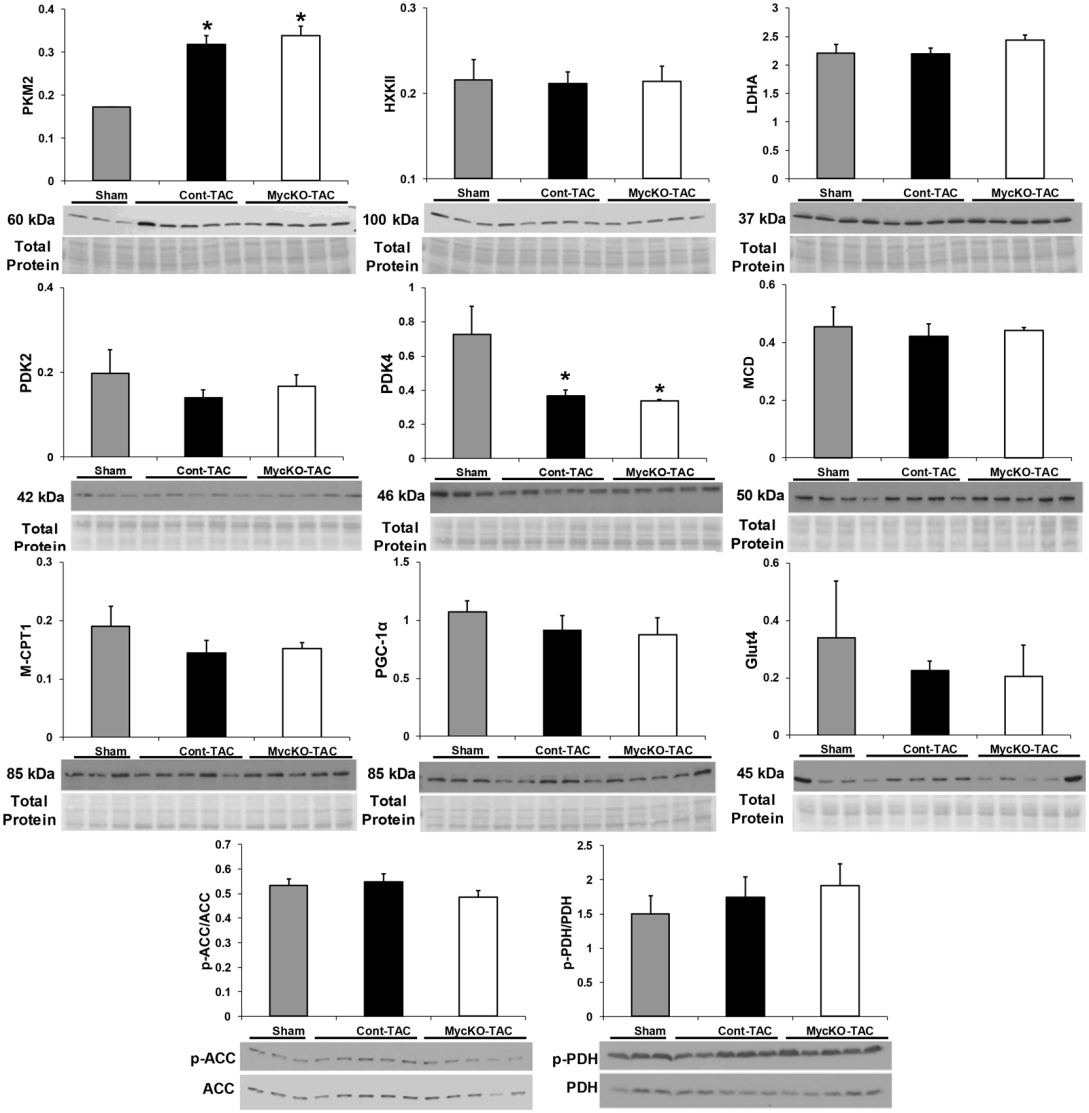
Expression of proteins involved in metabolism. All values are arbitrary units normalized to total protein levels (for phosphorylated proteins) or total protein staining as described in the methods. Molecular weights are noted. Values are means±SEM. PKM2, pyruvate kinase M2 isoform; LDHA, lactate dehydrogenase A; ACC, acetyl-CoA carboxylase; p-ACC, phospho-ACC; PDH, pyruvate dehydrogenase; p-PDH, phospho-PDH; HXKII, hexokinase II; MCD, malonyl-CoA carboxylase; M-CPT1, Muscle carnitine palmitoyltransferase 1; PGC-1α peroxisome proliferator-activated receptor gamma coactivator 1 alpha; Glut4, glucose transporter 4; PDK2, pyruvate dehydrogenase kinase 2; PDK4, pyruvate dehydrogenase kinase 4. For immunoblots, Sham is the Cont-Sham group. n = 3 Sham, n = 5 Cont-TAC and n = 5 MycKO-TAC. *p<0.05 between indicated group versus Sham.

We evaluated mechanisms which could explain our metabolic findings. Since substrate fractional contributions were similar between Cont-Sham and MycKO-Sham, only Cont-Sham was used in the immunoblots for comparison to TAC mice. Ahuja et al. found that Myc knockout during aortic constriction reduced RNA expression of LDHA and HXK2 [10]. Our results showed similar protein levels of both enzymes among the groups (Fig 3). We next assessed whether Myc transcriptionally regulates metabolism by altering protein levels of other key enzymes involved in carbohydrate and fatty acid metabolism. PDK4 protein levels were significantly greater in Sham compared to both Cont-TAC and MycKO-TAC; but were similar between Cont-TAC and MycKO-TAC (Fig 3). Total protein levels were also unchanged among the groups for a panel of other key regulatory metabolic enzymes (Fig 3). Carbohydrate oxidation is also regulated by PDH phosphorylation; whereas ACC phosphorylation affects fatty acid oxidation. Our assessment of both PDH and ACC phosphorylation also showed no differences (Fig 3). Given the lack of changes in these commonly assessed metabolic enzymes, we evaluated other potentially novel mechanisms including pyruvate kinase (PK) isoform switching, and protein posttranslational modifications by O-GlcNAc.

PK enzymatically converts phosphoenolpyruvate to pyruvate while yielding one molecule of ATP. PKM2 isoform expression is increased in proliferating cells, tumors, fetal/early post-natal heart, and failing human hearts [23–26]. Differing from PKM1, PKM2 enzyme activity is subject to allosteric inhibition. PKM2 inhibition decreases glucose oxidation in malignant cells. In tumors, Myc can promote alternative slicing of pre-mRNA to form the PKM2 isotype [27]. PKM2 protein levels significantly increased with TAC (p<0.05 Cont-Sham versus Cont-TAC and Cont-Sham versus MycKO-TAC); however this change was unaffected by Myc inhibition during aortic constriction (Fig 3).

**Fig 4:**
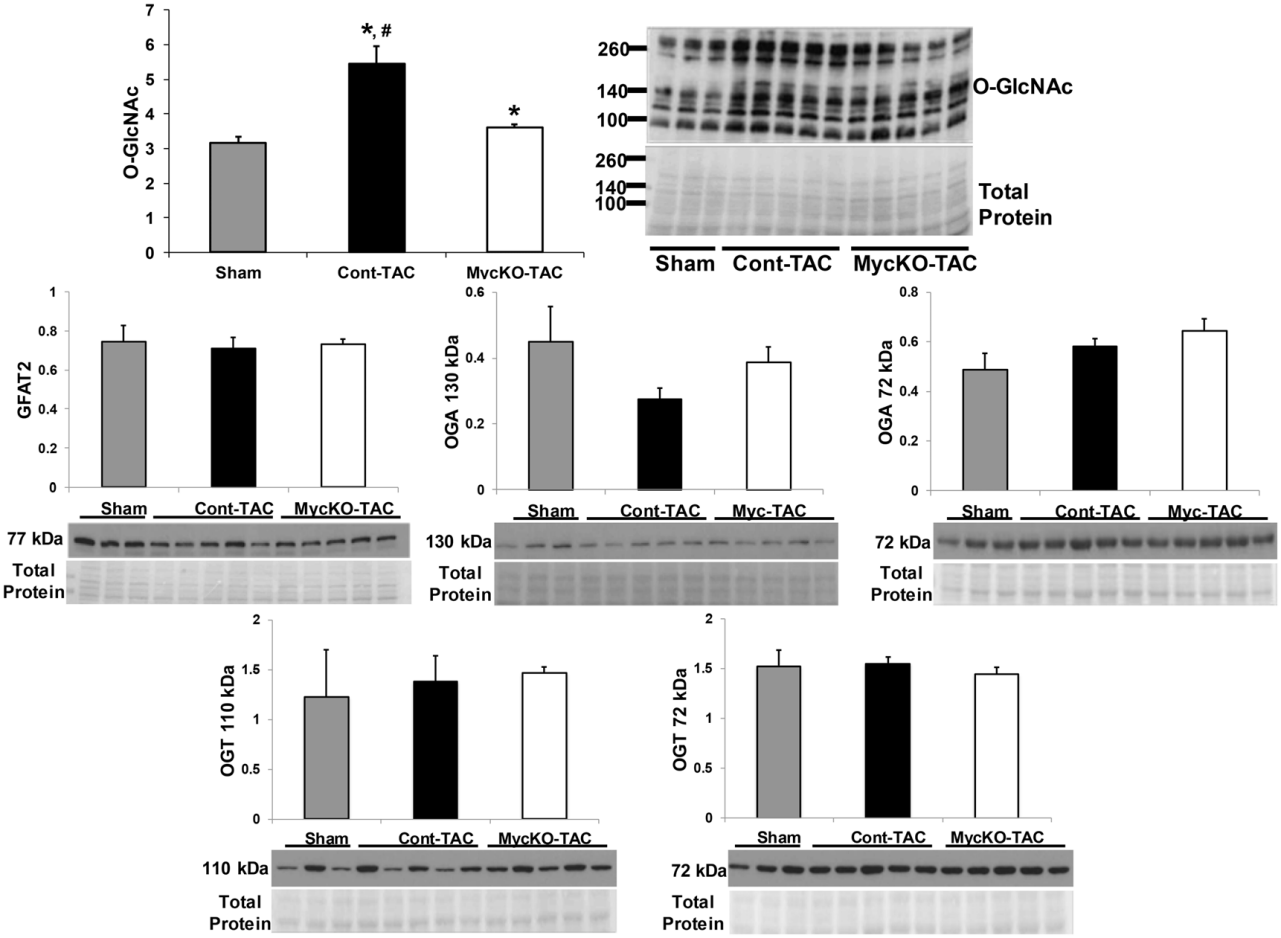
Total protein O-linked β-N-acetylglucosamine (O-GlcNAc) levels, and the regulatory enzymes glutamine-fructose-6-phosphate transaminase 2 (GFAT2), O-GlcNAc transferase (OGT), and O-GlcNAcase (OGA). Molecular weight markers (in kDa) for the O-GlcNAc western and protein staining are shown next to the respective images. OGT and OGA each had two bands, which corresponded to the molecular weights for known isoforms. Of note, the OGA western required different exposure times to identify both isoforms. Sham is the Cont-Sham group. n = 3 Sham, n = 5 Cont-TAC and n = 5 MycKO-TAC. * p<0.05 between the indicated group versus Sham, # p<0.05 Cont-TAC versus MycKO-TAC.

Our previous work showed that Myc activation increased myocardial total protein O-GlcNAc levels [11]. Therefore, we determined whether Myc knockout could affect protein function and metabolism during aortic constriction by altering O-GlcNAc posttranslational modifications. Global O-GlcNAc levels significantly increased in Cont-TAC compared to both Cont-Sham and MycKO-TAC (Fig 4, p<0.05 for both; S2 Table). There was also a small, but significant increase in O-GlcNAc levels in MycKO-TAC versus Sham (p<0.05).

We further determined whether the changes in O-GlcNAc levels were from alterations in protein levels of key enzymes regulating the O-GlcNAc pathway. The moiety necessary for O-GlcNAcylation is generated by the hexosamine biosynthesis pathway, an accessory pathway in the glycolytic breakdown of glucose to pyruvate. GFAT is the rate-limiting enzyme of the hexosamine biosynthesis pathway [28]. Once generated, the O-GlcNAc moiety is attached to proteins by the enzyme OGT. The enzyme OGA removes the O-GlcNAc moiety from proteins. There were no differences in protein levels for GFAT, OGT, and OGA among the experimental groups (Fig 4). Of note, OGT and OGA each had two bands, which corresponded to molecular weights of known isoforms of these proteins [29].

## Discussion

Our prior work demonstrated that Myc-induced cardiac hypertrophy increased fatty acid oxidation while maintaining compensated function in the unstressed heart [11]. Nearly all types of hypertrophic stimuli promote Myc transcription; however, it was previously unknown whether Myc had similar effects during hypertrophic stressors such as aortic constriction. In the current study, we evaluated substrate metabolism as a mechanism whereby Myc modifies the response to pressure overload hypertrophy hypertrophic response.

### Cardiac function and hypertrophy

Clinically, the degree of hypertrophy from pressure overload correlates with the Doppler estimated mean pressure gradient at the site of flow obstruction, usually the aortic valve or coarctation of the aorta. However, few previous metabolic studies utilizing TAC have provided data regarding the pressure gradient across the constriction site [30–34]. We used Doppler measurements and obtained similar estimated mean pressure gradients of about 43 mmHg between Cont-TAC and MycKO-TAC. Comparable pressure gradients in humans warrant interventional relief of the obstruction to prevent progression to uncompensated cardiac hypertrophy. Thus, we achieved and documented consistent hypertrophic stimuli between these groups, and eliminated the unknown variability in TAC prevalent in other studies.

Prior studies employing TAC in these inducible Myc knockout mice showed decreased cardiac mass, cardiomyocyte size, and hypertrophic signaling via atrial natriuretic peptide mRNA [9,10]. The pressure gradient was not reported in these studies, so we confirmed that Myc knockout reduced hypertrophy by demonstrating decreased cardiac mass in MycKO-TAC versus Cont-TAC. Limited functional analysis in these previous studies also demonstrated maintenance of cardiac function in Myc knockout mice after TAC [9]. More detailed analyses in the current study, including *ex vivo* functional assessments, showed no functional impairment from Myc knockout during early aortic constriction. In fact, the data trends for dP/dT suggest that Myc expression may adversely affect both systolic and diastolic function. Further studies with even longer TAC periods are required to determine the eventual functional outcome, and whether Myc shortens the time to decompensation.

### Substrate preferences for the citric acid cycle during pressure overload hypertrophy

Prevailing theory suggests that a metabolic shift towards glucose or overall carbohydrate oxidation promotes cardiac compensation during cardiac hypertrophy [13,14,35]. This theory is based the higher oxygen efficiency of ATP generated from glucose [36]. Some studies demonstrate contradictory data showing a requirement for metabolic shift towards fatty acid oxidation for maintenance of cardiac function during pressure overload hypertrophy [20]. Overall, many of these interpretations are based on data derived from constitutive forced expression or knockout of specific downstream regulators of cardiac metabolism, such as PGC-1α, ACC or GLUT1 [20,30,32–34].

In our study, we employed a novel approach by using an inducible cardiac knockout of a proto-oncogene expressed during hypertrophy, but not during normal physiology conditions [10]. Our metabolic results are notable for two findings. First, we demonstrated that Myc alters oxidative substrate preferences during hypertrophy. Myc knockout reduced fatty acid and ketone fractional contribution for the citric acid cycle with a reciprocal increase in unlabeled (majority glucose) utilization during early TAC. These metabolic changes were not present in Sham hearts, indicating that Myc does not regulate oxidative metabolism during normal physiologic conditions. Secondly, the metabolic changes from Myc were not necessary to maintain contractile function. Thus, augmenting fatty acid contribution to the citric acid cycle did not promote cardiac compensation at least over the short-term. Our findings do not eliminate that possibility that metabolic perturbation have important functional effects when employed over a longer duration or during heart failure.

Our substrate preference results seemingly differ from the commonly described metabolic changes during hypertrophy/heart failure which include decreased fatty acid oxidation and increased glucose utilization [12]. However, recent findings by Zhabyeyev et al. are consistent with our data [31]. Zhabyeyev et al. found maintained fatty acid oxidation and reduced glucose oxidation in mice after one month of TAC [30]. Thus, the fractional contribution from fatty acids to the citric acid cycle increased in their study due to the decline in glucose oxidation. All told, the metabolic changes during hypertrophy/heart failure appear more variable than commonly assumed. Cardiac metabolism is likely influenced by the animal/strain used, hypertrophic stimuli and the degree of hypertrophy or heart failure. Further, perfusion differences in the method (retrograde versus antegrade coronary perfusion), included substrates, substrate concentrations, degree of afterload, insulin inclusion and insulin concentration can affect results and comparisons among metabolic studies [20,30,31].

### Molecular mechanisms for Myc’s regulation of substrate utilization

Previous work showed that Myc knockout for a similar TAC duration decreased LDHA and HXK2 RNA levels [10]. Theoretically, decreased LDHA could reduce the conversion of glucose to lactate and thereby promote glucose oxidation. However, we could detect no differences in protein levels of these enzymes between groups using semi-quantitative immunoblots. Potentially there are small, but physiologically important changes in LDHA and/or other metabolic enzymes that are below the detection limits of immunoblots. We also did not identify changes in protein levels of other important and commonly evaluated metabolic enzymes including phosphorylation of ACC and PDH. Accordingly, Myc may regulate substrate preferences by altering expression of other proteins and/or through non-transcriptional mechanisms. In this context, we investigated two potentially novel mechanisms of metabolic regulation.

Our study suggests a relationship between aortic constriction and increased protein levels of PKM2. Rees et al. recently demonstrated increased PKM2 expression in failing human hearts, which was partially reversed by mechanical unloading therapy (ventricular assist devices) [26]. As part of their investigation, mice treated with sunitinib (a tyrosine kinase inhibitor that increases glucose metabolism) had increased myocardial PKM2 and Myc transcription. Thus, they speculated that Myc may plan role in PKM2 induction [26]. In the current study, PKM2 protein levels were not linked to Myc expression. Further, our results show that Myc does not regulate substrate preferences during TAC by altering overall PKM2 levels. PKM2 is subject to allosteric regulation, with dimeric PKM2 inhibiting glucose oxidative metabolism [23,24,37]. Since immunoblots cannot determine the PKM2 configuration, Myc could potentially regulate substrate preferences through allosteric modifications of PKM2.

Myc knockout reduced global O-GlcNAc levels during pressure overload hypertrophy. Laczy et al. found that acute O-GlcNAcylation promoted fatty acid oxidation [15]. They perfused isolated rat hearts with glucosamine to increase total protein O-GlcNAcylation. Glucosamine caused a dose dependent increase in O-GlcNAc levels that was associated with increased palmitate oxidation. Based upon Laczy et al.’s study, the changes in O-GlcNAc levels between Cont-TAC and MycKO-TAC could potentially cause the differences in fatty acid fractional contributions. Additional experiments with direct O-GlcNAc manipulation during aortic constriction are warranted to prove whether this posttranslational modification regulates substrate metabolism during hypertrophy. Since no site-specific antibodies for O-GlcNAc modified proteins are commercially available, it has also proven challenging to identify specific metabolic proteins subject to O-GlcNAcylation. O-GlcNAc’s effect on cardiac metabolism represents an important area for future studies.

Myc knockout did not affect protein levels of the key O-GlcNAc regulatory enzymes GFAT, OGT or OGA. Although Myc commonly acts as a transcription factor, our results suggest that Myc alters O-GlcNAc levels through non-transcriptional mechanisms. These mechanisms potentially include changes in GFAT, OGT and/or OGA activity by post-translational modifications. Additionally, generation of the O-GlcNAc moiety depends on shuttling glucose into the hexosamine biosynthesis pathway; an accessary pathway from glycolysis. Although O-GlcNAc may influence substrate preferences; paradoxically, metabolic changes could also influence O-GlcNAc levels by affecting hexosamine biosynthesis flux [38]. The integration between substrate metabolism and the hexosamine biosynthesis pathway requires further clarifications, particularly during cardiac hypertrophy.

### Limitations

Although we tried to limit atmospheric exposure, the oxygen tension in the coronary effluent is potentially altered by atmospheric gas exchange. This limitation would affect the oxygen consumption values used to calculate estimated flux for overall citric acid cycle and individual substrates. Accordingly, the flux values should be considered calculated estimated values. However, individual substrate fractional contributions to the citric acid cycle are not affected by this limitation.

Hypertrophy often affects non-oxidative glycolysis; however, the MRS based method used in this study does not measure glycolytic flux. Thus, we cannot make conclusions on Myc’s affect for overall glucose utilization during hypertrophy.

## Conclusion

The current study demonstrates that Myc affects myocardial substrate utilization preferences for the citric acid cycle during early pressure overload hypertrophy, but not during normal physiology. However, Myc’s regulation of metabolic changes was not necessary to maintain cardiac function. Myc knockout altered global O-GlcNAc levels during aortic constriction, which may mechanistically regulate the changes in substrate preferences.

## Supporting Information

S1 Table Individual experiment fractional contributions to the citric acid cycle for unlabeled substrates (unlabeled), acetoacetate as ketone bodies (AcAC and/or ketone), lactate, and free fatty acids (FFA) in the hearts subjected to transverse aortic constriction (TAC) and calculated estimated flux values. Flux units are μmol/g/min. Acetoacetate was used for ketones. Note: Sample 042213A was excluded for flux value calculations because of a perfusate leak from the left atrium, which would render the MVO2 value inaccurate. However, the leak does not affect the fractional contribution values.
(XLSX)

S2 Table Individual O-GlcNAc western blot quantification values. The O-GlcNAc western blot and total protein quantification pictures are shown in manuscript Fig 4.
(XLSX)
